# LIFESTYLE PHYSICAL ACTIVITY IN OLDER WOMEN: ASSOCIATIONS OF CHANGE, SELF-EFFICACY, AND WELL-BEING

**DOI:** 10.1093/geroni/igz038.609

**Published:** 2019-11-08

**Authors:** Leanne L Lefler, Shelly Y Lensing, Kimberly K Garner

**Affiliations:** 1 University of Arkansas for Medical Sciences, Arkansas, United States; 2 University of Arkansas for Medical Sciences, Little Rock, Arkansas, United States; 3 Geriatric Research, Education and Clinical Center, Central Arkansas Veterans Healthcare, North Little Rock, Arkansas, United States

## Abstract

Reduction of cardiovascular disease risk in undeserved populations, such as older women, is a top priority of the U.S. Our innovative trial tested a new approach to PA promotion for older women—motivational interviewing (MI), shifting the paradigm from structured exercise to self-selected activities. We present data comparing stage of change (SOC), self-efficacy for exercise (SEE), and well-being: 8 dimensions (physical, social, role limitations, emotional, general mental health, vitality, health perceptions and pain) and associations with physical activity outcomes in the Lifestyle Physical Activity for Women (LPAW) clinical trial. Methods: 106 women, > 60 years old, who did not engage in regular PA, and were not frail, participated in a clinical trial of a tailored MI intervention to increase PA. We report baseline, 3 and 6 month repeated measures and PA associations with SOC, SEE, and well-being (SF36). Results: Of 106 women, 36% were Black and 63% White, with a mean age of 69. Significant improvement in SOC in both arms noted but the proportion in action/maintenance was significantly higher in the PA arm at 3 mos (78% vs. 55%, P=0.045) and 6 mos (79% vs. 50%, P= 0.019). A decrease in SEE for control (p=.001), but not for PA arm (p=.45); at 6 months, The PA arm had greater SEE compared to control. There were significant arm difference for physical component scores of SF36 (p=.02), but not for mental scores. Associations with PA will be tabulated. Conclusions: Preliminary results support the PA intervention, more data to be presented.

